# Supplementary material for: The Relationship Between Reduced Hand Dexterity and Brain Structure Abnormality in Older Adults
Source: Geriatrics (Basel). 2024 Dec 17;9(6):165. doi: 10.3390/geriatrics9060165 (PMC11728121; doi:10.3390/geriatrics9060165)
Supplement: Supplementary file 1 [file geriatrics-09-00165-s001.zip › geriatrics-3338314-supplementary.pdf]

geriatrics

Article

The Relationship Between Reduced Hand Dexterity and Brain Structure Abnormality in Older Adults

SUPPLEMENTAL MATERIALS

Table S1. Correlations between hand dexterity, demographics, clinical, and brain measures

Variable 1	Variable 2	Correlation coefficient	*p*-value	Hand for which elastic net model was conducted Dominant hand = right. Non-dominant hand = left.
**Correlations between clinical and hand dexterity variables**				
**9HPT dominant hand**	**9HPT non-dominant hand**	**0.66**	**0.002**	
**MoCA**	**IQ**	**0.49**	**0.03**	
Age	IQ	-0.36	n.s.	
Age	MoCA	-0.27	n.s.	
9HPT non-dominant hand	Age	0.37	n.s.	
9HPT non-dominant hand	IQ	-0.19	n.s.	
9HPT non-dominant hand	MoCA	0.02	n.s.	
9HPT dominant hand	Age	0.18	n.s.	
9HPT dominant hand	IQ	0.01	n.s.	
9HPT dominant hand	MoCA	0.24	n.s.	
**Correlation of clinical and dexterity variables with brain variables**				
**9HPT non-dominant hand**	**Right Caudate**	**0.67**	**0.001**	**left**
**9HPT non-dominant hand**	**Left IP2**	**-0.64**	**0.002**	**left**
**9HPT non-dominant hand**	**Right d23ab**	**0.65**	**0.002**	**left**
**9HPT non-dominant hand**	**Left FOP1**	**-0.61**	**0.005**	**both**
**9HPT dominant hand**	**Right IFJa**	**-0.6**	**0.005**	**right**
**IQ**	**Right Caudate**	**-0.58**	**0.007**	**left**
**Age**	**Left IP2**	**-0.57**	**0.008**	**left**
**9HPT non-dominant hand**	**Left choroid plexus**	**0.58**	**0.008**	**both**
**Age**	**Left LBelt**	**-0.56**	**0.01**	**right**
**9HPT dominant hand**	**Left VMV3**	**-0.55**	**0.01**	**right**
**9HPT non-dominant hand**	**Left VMV3**	**-0.54**	**0.01**	**right**
**9HPT dominant hand**	**Right TPOJ2**	**-0.54**	**0.01**	**right**
**9HPT dominant hand**	**Left LO1**	**-0.51**	**0.02**	**right**
**MoCA**	**Right TPOJ3**	**0.5**	**0.03**	**right**
**9HPT dominant hand**	**Right STGa**	**-0.5**	**0.03**	**right**

**9HPT non-dominant hand**	**Right IFJa**	**-0.49**	**0.03**	**right**
**9HPT dominant hand**	**Left FOP1**	**-0.49**	**0.03**	**both**
**9HPT dominant hand**	**Left TGd**	**0.49**	**0.03**	**right**
**9HPT non-dominant hand**	**Left TGd**	**0.48**	**0.03**	**right**
**9HPT dominant hand**	**Left LBelt**	**-0.48**	**0.03**	**right**
**9HPT dominant hand**	**Right LIPv**	**-0.45**	**0.04**	**right**
**9HPT dominant hand**	**Right TPOJ3**	**0.46**	**0.04**	**right**
Age	Left 6mp	0.09	n.s.	right
Age	Left FOP1	-0.22	n.s.	both
Age	Left IP0	0.19	n.s.	right
Age	Left LO1	-0.13	n.s.	right
Age	Left TGd	0.11	n.s.	right
Age	Left VMV3	-0.21	n.s.	right
Age	Left choroid plexus	0.18	n.s.	both
Age	Left Thalamus	-0.44	n.s.	right
Age	Right d23ab	0.23	n.s.	left
Age	Right IFJa	-0.17	n.s.	right
Age	Right LIPv	-0.17	n.s.	right
Age	Right STGa	0.11	n.s.	right
Age	Right TPOJ2	-0.18	n.s.	right
Age	Right TPOJ3	-0.44	n.s.	right
Age	Right V3B	-0.2	n.s.	right
Age	Right Caudate	0.44	n.s.	left
IQ	Left 6mp	0.13	n.s.	right
IQ	Left FOP1	0.05	n.s.	both
IQ	Left IP0	-0.15	n.s.	right
IQ	Left IP2	0.4	n.s.	left
IQ	Left LBelt	0.07	n.s.	right
IQ	Left LO1	-0.05	n.s.	right
IQ	Left TGd	0.21	n.s.	right
IQ	Left VMV3	0.24	n.s.	right
IQ	Left choroid plexus	0.36	n.s.	both
IQ	Left Thalamus	-0.27	n.s.	right
IQ	Right d23ab	-0.3	n.s.	left
IQ	Right IFJa	0.1	n.s.	right
IQ	Right LIPv	-0.12	n.s.	right
IQ	Right STGa	0.11	n.s.	right
IQ	Right TPOJ2	0.01	n.s.	right
IQ	Right TPOJ3	0.36	n.s.	right
IQ	Right V3B	0.18	n.s.	right
9HPT non-dominant hand	Left 6mp	0.02	n.s.	right

9HPT non-dominant hand	Left IP0	-0.05	n.s.	right
9HPT non-dominant hand	Left LBelt	-0.42	n.s.	right
9HPT non-dominant hand	Left LO1	-0.19	n.s.	right
9HPT non-dominant hand	Left Thalamus	-0.22	n.s.	right
9HPT non-dominant hand	Right LIPv	0.13	n.s.	right
9HPT non-dominant hand	Right STGa	-0.22	n.s.	right
9HPT non-dominant hand	Right TPOJ2	-0.3	n.s.	right
9HPT non-dominant hand	Right TPOJ3	0.29	n.s.	right
9HPT non-dominant hand	Right V3B	-0.24	n.s.	right
MoCA	Left 6mp	0.08	n.s.	right
MoCA	Left FOP1	0.05	n.s.	both
MoCA	Left IP0	-0.22	n.s.	right
MoCA	Left IP2	0.39	n.s.	left
MoCA	Left LBelt	0	n.s.	right
MoCA	Left LO1	-0.08	n.s.	right
MoCA	Left TGd	0.04	n.s.	right
MoCA	Left VMV3	-0.13	n.s.	right
MoCA	Left choroid plexus	0.43	n.s.	both
MoCA	Left Thalamus	0.2	n.s.	right
MoCA	Right d23ab	0.03	n.s.	left
MoCA	Right IFJa	-0.42	n.s.	right
MoCA	Right LIPv	-0.25	n.s.	right
MoCA	Right STGa	0.02	n.s.	right
MoCA	Right TPOJ2	-0.23	n.s.	right
MoCA	Right V3B	0.33	n.s.	right
MoCA	Right.Caudate_etiv	-0.04	n.s.	left
9HPT dominant hand	Left 6mp	0.4	n.s.	right
9HPT dominant hand	Left IP0	-0.42	n.s.	right
9HPT dominant hand	Left IP2	-0.39	n.s.	left
9HPT dominant hand	Left choroid plexus	0.4	n.s.	both
9HPT dominant hand	Left Thalamus	-0.36	n.s.	right
9HPT dominant hand	Right d23ab	0.23	n.s.	left
9HPT dominant hand	Right V3B	-0.41	n.s.	right
9HPT dominant hand	Right.Caudate_etiv	0.37	n.s.	left
**Correlation between brain variables**				
**Right TPOJ2**	**Left LO1**	**0.61**	**0.004**	**right**
**Right IFJa**	**Left VMV3**	**0.55**	**0.01**	**right**
**Right d23ab**	**Left IP2**	**-0.55**	**0.01**	**left**
**Left TGd**	**Left LBelt**	**-0.54**	**0.02**	**right**
**Left choroid plexus**	**Right IFJa**	**-0.52**	**0.02**	**right**
**Right Caudate**	**Right d23ab**	**0.52**	**0.02**	**left**

**Left FOP1**	**Left LBelt**	**0.51**	**0.02**	**right**
**Left 6mp**	**Left LBelt**	**-0.49**	**0.03**	**right**
**Right LIPv**	**Left 6mp**	**-0.48**	**0.03**	**right**
**Right Caudate**	**Left IP2**	**-0.45**	**0.04**	**left**
**Right d23ab**	**Left FOP1**	**-0.45**	**0.05**	**left**
**Left Thalamus**	**Left LO1**	**0.45**	**0.05**	**right**
Left 6mp	Left FOP1	-0.27	n.s.	right
Left 6mp	Left IP0	-0.26	n.s.	right
Left 6mp	Left TGd	0.09	n.s.	right
Left 6mp	Left VMV3	-0.42	n.s.	right
Left FOP1	Left IP0	0.15	n.s.	right
Left FOP1	Left IP2	0.4	n.s.	left
Left FOP1	Left TGd	-0.44	n.s.	right
Left FOP1	Left VMV3	0.16	n.s.	right
Left IP0	Left LBelt	0.11	n.s.	right
Left IP0	Left VMV3	0.13	n.s.	right
Left LO1	Left 6mp	-0.28	n.s.	right
Left LO1	Left FOP1	0.05	n.s.	right
Left LO1	Left IP0	0.12	n.s.	right
Left LO1	Left LBelt	0	n.s.	right
Left LO1	Left TGd	-0.01	n.s.	right
Left LO1	Left VMV3	0.17	n.s.	right
Left TGd	Left IP0	0.06	n.s.	right
Left TGd	Left VMV3	-0.24	n.s.	right
Left VMV3	Left LBelt	0.37	n.s.	right
Left choroid plexus	Left 6mp	0.2	n.s.	right
Left choroid plexus	Left FOP1	-0.36	n.s.	both
Left choroid plexus	Left IP0	-0.29	n.s.	right
Left choroid plexus	Left IP2	-0.2	n.s.	left
Left choroid plexus	Left LBelt	-0.33	n.s.	right
Left choroid plexus	Left LO1	-0.08	n.s.	right
Left choroid plexus	Left TGd	0.37	n.s.	right
Left choroid plexus	Left VMV3	-0.25	n.s.	right
Left choroid plexus	Right d23ab	0.41	n.s.	left
Left choroid plexus	Right LIPv	-0.11	n.s.	right
Left choroid plexus	Right STGa	0.12	n.s.	right
Left choroid plexus	Right TPOJ2	-0.01	n.s.	right
Left choroid plexus	Right TPOJ3	0.24	n.s.	right
Left choroid plexus	Right V3B	0	n.s.	right
Left choroid plexus	Right Caudate	0.37	n.s.	left
Left Thalamus	Left 6mp	-0.31	n.s.	right

Left Thalamus	Left FOP1	0.15	n.s.	right
Left Thalamus	Left IP0	0.22	n.s.	right
Left Thalamus	Left LBelt	0.42	n.s.	right
Left Thalamus	Left TGd	-0.32	n.s.	right
Left Thalamus	Left VMV3	0.08	n.s.	right
Left Thalamus	Left choroid plexus	-0.33	n.s.	right
Left Thalamus	Right IFJa	-0.14	n.s.	right
Left Thalamus	Right LIPv	0.18	n.s.	right
Left Thalamus	Right STGa	-0.08	n.s.	right
Left Thalamus	Right TPOJ2	0.27	n.s.	right
Left Thalamus	Right TPOJ3	-0.08	n.s.	right
Left Thalamus	Right V3B	0.3	n.s.	right
Right IFJa	Left 6mp	-0.22	n.s.	right
Right IFJa	Left FOP1	0.3	n.s.	right
Right IFJa	Left IP0	0.4	n.s.	right
Right IFJa	Left LBelt	0.36	n.s.	right
Right IFJa	Left LO1	0.05	n.s.	right
Right IFJa	Left TGd	-0.19	n.s.	right
Right IFJa	Right STGa	0.13	n.s.	right
Right IFJa	Right TPOJ2	0.16	n.s.	right
Right IFJa	Right TPOJ3	-0.25	n.s.	right
Right LIPv	Left FOP1	0.04	n.s.	right
Right LIPv	Left IP0	0.17	n.s.	right
Right LIPv	Left LBelt	0.42	n.s.	right
Right LIPv	Left LO1	0.24	n.s.	right
Right LIPv	Left TGd	-0.27	n.s.	right
Right LIPv	Left VMV3	-0.01	n.s.	right
Right LIPv	Right IFJa	0.28	n.s.	right
Right LIPv	Right STGa	0.43	n.s.	right
Right LIPv	Right TPOJ2	0.08	n.s.	right
Right LIPv	Right TPOJ3	0.1	n.s.	right
Right STGa	Left 6mp	0.01	n.s.	right
Right STGa	Left FOP1	0.09	n.s.	right
Right STGa	Left IP0	0.14	n.s.	right
Right STGa	Left LBelt	0.13	n.s.	right
Right STGa	Left LO1	-0.09	n.s.	right
Right STGa	Left TGd	-0.27	n.s.	right
Right STGa	Left VMV3	0.09	n.s.	right
Right STGa	Right TPOJ2	0.07	n.s.	right
Right STGa	Right TPOJ3	-0.06	n.s.	right
Right TPOJ2	Left 6mp	0.16	n.s.	right

Right TPOJ2	Left FOP1	0.24	n.s.	right
Right TPOJ2	Left IP0	0.32	n.s.	right
Right TPOJ2	Left LBelt	0.05	n.s.	right
Right TPOJ2	Left TGd	-0.08	n.s.	right
Right TPOJ2	Left VMV3	0.06	n.s.	right
Right TPOJ2	Right TPOJ3	-0.24	n.s.	right
Right TPOJ3	Left 6mp	0.28	n.s.	right
Right TPOJ3	Left FOP1	-0.13	n.s.	right
Right TPOJ3	Left IP0	-0.28	n.s.	right
Right TPOJ3	Left LBelt	0	n.s.	right
Right TPOJ3	Left LO1	-0.32	n.s.	right
Right TPOJ3	Left TGd	0.2	n.s.	right
Right TPOJ3	Left VMV3	-0.31	n.s.	right
Right V3B	Left 6mp	-0.03	n.s.	right
Right V3B	Left FOP1	0.3	n.s.	right
Right V3B	Left IP0	0.33	n.s.	right
Right V3B	Left LBelt	0.21	n.s.	right
Right V3B	Left LO1	0.04	n.s.	right
Right V3B	Left TGd	-0.12	n.s.	right
Right V3B	Left VMV3	0.15	n.s.	right
Right V3B	Right IFJa	0.21	n.s.	right
Right V3B	Right LIPv	0.03	n.s.	right
Right V3B	Right STGa	0.23	n.s.	right
Right V3B	Right TPOJ2	0.26	n.s.	right
Right V3B	Right TPOJ3	0.13	n.s.	right
Right Caudate	Left FOP1	-0.37	n.s.	left

Note: The abbreviation "n.s." stands for not significant results ($p < 0.05$)
